# Characterization of the mutational status of glioblastoma and high-grade astrocytomas in a Latin American cohort

**DOI:** 10.1038/s41598-025-06129-z

**Published:** 2025-10-03

**Authors:** Rodrigo Fernández-Gajardo, Hery Urra, Mauricio Sáez, Philippe Pihán, Beatriz Fonseca, Carolina Sanchez-Doñas, Gabriel Cavada, Claudia Tissera, Rómulo Melo, David Rojas-Zalazar, José M. Matamala, Claudio Hetz

**Affiliations:** 1 Program of Cellular and Molecular Biology, Institute of Biomedical Sciences, University of Chile, 1027 Independencia Av., Santiago, Chile; 2 Biomedical Neuroscience Institute (BNI), Faculty of Medicine, University of Chile, Santiago, Chile; 3 Department of Neurological Sciences, Faculty of Medicine, University of Chile, Santiago, Chile; 4 Department of Neurosurgery, Clínica Santa María, Santiago, Chile; 5 FONDAP Center for Geroscience, Brain Health, and Metabolism (GERO), Santiago, Chile; 6 Facultad de Odontología, Universidad San Sebastián, Bellavista, Santiago, Chile; 7 Department of Diagnostic and Evaluation Processes, Faculty of Health Sciences, Universidad Católica de Temuco, Temuco, Chile; 8 Advanced Genomics Core, Universidad Mayor, Santiago, Chile; 9 Public Health Department, Faculty of medicine, University of Chile, Santiago, Chile; 10 Pathology Department, Asenjo Neurosurgery Institute, Santiago, Chile; 11 Buck Institute for Research on Aging, Novato, CA 94945 USA; 12 Institute of Biomedical Sciences, University of Chile, 486 Salvador Av., Santiago, Chile

**Keywords:** Genomic epidemiology, Underrepresented populations, Next-generation sequencing, Glioblastoma, Precision medicine, Population genetics, CNS cancer, Genetics research, Epidemiology

## Abstract

High-grade astrocytomas vary in incidence and mortality across populations, with Hispanic and Latino groups largely underrepresented in genomic epidemiology studies. This study characterizes the presence of known mutations of high-grade astrocytomas in a Latin American cohort through targeted genomic analysis of 70 Chilean patients. Molecular markers, including *IDH*, *TERTp*, *H3*, *TP53*, *PTEN*, *EGFR*, and *CDKN2A*, were assessed alongside survival analyses. Our results mostly aligned with international cohorts, confirming the importance of established molecular markers in glioblastoma. Novel damaging *TP53* and *PTEN* mutations were identified, expanding the genetic spectrum of known mutations for these genes, while a lower-than-expected *NF1* mutation frequency was observed (*p* < 0.01). These findings highlight the importance of examining underrepresented populations, providing insights into the molecular characteristics of high-grade astrocytomas in Latin America. Our findings contribute to understanding the diversity of genomic features across astrocytoma populations, setting a foundation for future international comparative studies.

**Supplementary Information:**

The online version contains supplementary material available at 10.1038/s41598-025-06129-z.

## Introduction

High-grade astrocytomas (HGA) are a subset of aggressive and lethal brain cancers that constitute a significant challenge in oncology. A comprehensive understanding of the molecular mechanisms that drive HGA is crucial for the development of effective diagnostic and therapeutic approaches. Although extensive research has been conducted on HGA across diverse populations worldwide^1–5^, a notable knowledge gap exists concerning the incidence of mutations associated with HGA in Latin American populations. For example, in the largest cohort of patients with HGA and genomic data currently available, the TCGA pan-cancer atlas glioblastoma database, only approximately 2% are individuals of Hispanic or Latino ethnicity^1^.

Latin American ancestry refers to a highly admixed genetic background, as previously demonstrated in the Chilean population and other South American countries^6^. Genomic ancestry studies have shown an average genetic composition of approximately **40% Native American**,** 55% European**,** and 2% African ancestry**^7–9^while studies on uniparental inheritance have reported up to **88% Native American mitochondrial DNA** in populations from different Chilean cities^10,11^a pattern also observed in other regions of South America^7^.

With the increasing integration of molecular criteria into the widely used WHO classification of tumors of the central nervous system (CNS), the importance of accurate and reliable molecular techniques has increased in clinical practice^12^. Molecular diagnostics using next-generation sequencing (NGS)-based methods have become the gold standard for diagnostic workups^12^. The current criteria for diffuse high-grade astrocytoma classification include disease-defining alterations such as isocitrate dehydrogenase 1/2 (*IDH*) mutations and histone 3 (*H3*) gene family (*H3F3A*, *H3C2*) mutations, as well as grade-defining alterations such as telomerase reverse transcriptase promoter (*TERTp*) mutations, epidermal growth factor receptor (*EGFR*) gene amplification and the combined gain of entire chromosome 7 and loss of entire chromosome 10 in the case of *IDH*-wildtype tumors (glioblastoma), or cyclin-dependent kinase inhibitor 2 A/B (*CDKN2A/B*) deletion in the case of *IDH*-mutant tumors (astrocytoma, *IDH*-mutant)^13^. Molecular classification profoundly influences the prognosis and survival of glioblastoma patients, directly impacting their treatment, follow-up, and care^14–17^.

Here, we investigated the mutational status of known molecular drivers of HGA in a Latin American cohort, focusing on Chile as a nation characterized by its diverse ethnic composition, including Indigenous, European, and African influences^18^. By comparing the mutation frequencies in our cohort with those reported in international studies, we seek to determine the relevance of existing knowledge and treatment approaches for Latin American patients. This study aimed to provide insights into the genetic drivers of HGA in a South American population and underscore the broader importance of studying ethnic-specific genetic variations and their association with clinical outcomes.

## Methods

### Patients

We conducted an observational retrospective cohort study that included 70 patients aged 18 years and above with a confirmed diagnosis of glioblastoma (*IDH*-wildtype or *IDH*-mutant) or anaplastic astrocytoma, classified according to the WHO CNS tumor classification system in place at the time of each patient’s diagnosis. Patients who underwent total or partial resection surgery for a newly diagnosed high-grade astrocytoma at the Asenjo Neurosurgery Institute between January 2014 and January 2020 were included in this study. Patients with a previous diagnosis of diffuse glioma, incomplete follow-up, a positive fluorescence in situ hibridization for 1p/19q codeletion and/or insufficient tumor tissue samples for molecular studies were excluded. Additionally, five patients who underwent anterior temporal lobectomy for epilepsy treatment were included in the analysis as non-tumor tissue controls. A specific informed consent was obtained from all patients and controls. This study was approved by the Ethics Committee of the Metropolitan East Health Service in October 2017, and it was conducted in accordance with the principles of the Declaration of Helsinki.

### Panel design

We utilized the AmpliSeq™ for Illumina tool to create a customized amplicon panel covering specific genomic regions of interest of several neuro-oncology-related genes. We used databases such as cBioPortal, NCBI/Clinvar, and the Catalogue of Somatic Mutations in Cancer (COSMIC) to identify relevant genomic coordinates. Amplicons were then designed to cover these mutations (see Supplementary Table 1). A coverage depth of greater than 200 reads per position of interest was planned to detect allelic frequencies as low as 5%, with a threshold of ≥ 10 altered reads, following literature recommendations^19^. For detailed methods, see **Supplementary methods**.

## Results

### Clinical variables

The study cohort included 70 patients diagnosed with HGA. All patients reported a “Latin American” ancestry, and no patients reported a purely indigenous, European or other ancestries. The most relevant clinical variables, including access to different treatments and outcomes for all patients, are presented in Table 1.

**Table 1:** Baseline characteristics of 70 high-grade Astrocytoma patients.

Clinical characteristics	
Age, mean (sd, range)	57.8 years (11.9, 24–76)
Sex (M: W)	36:34
Hypertension, n (%)	33/70 (47.1%)
Diabetes, n (%)	10/70 (14.3%)
Smoking, n (%)	13/70 (18.6%)
Grade III, n (%)	3/70 (4.3%)
Grade IV, n (%)	67/70 (95.7%)
Tumor location	
Cortical/subcortical	59/70 (84.3%)
Deep-seated	8/70 (11.4%)
Multicentric	3/70 (4.3%)
Extent of resection	
Total resection	44/70 (62.9%)
Subtotal resection	26/70 (37.1%)
Adjuvant therapy	
RT alone, n (%)	16/70 (22.9%)
RT + temozolomide, n (%)	14/70 (20%)
None or paliative care	40/70 (57.1%)
Positive R132H IHC	7/38
30-day postoperative KPS, median (IQR)	80 (60–90)
Dead during follow-up	70/70 (100%)
Overall survival, median (IQR)	8.3 months (3.9–18.0)

Demographics, comorbidities, tumor characteristics, therapies and clinical outcomes are shown.

*RT* radiotherapy, *KPS* Karnosfsky performance score, *IQR* Interquartile range.

### Frequency of the most common genomic alterations in HGA

**Fig. 1:**
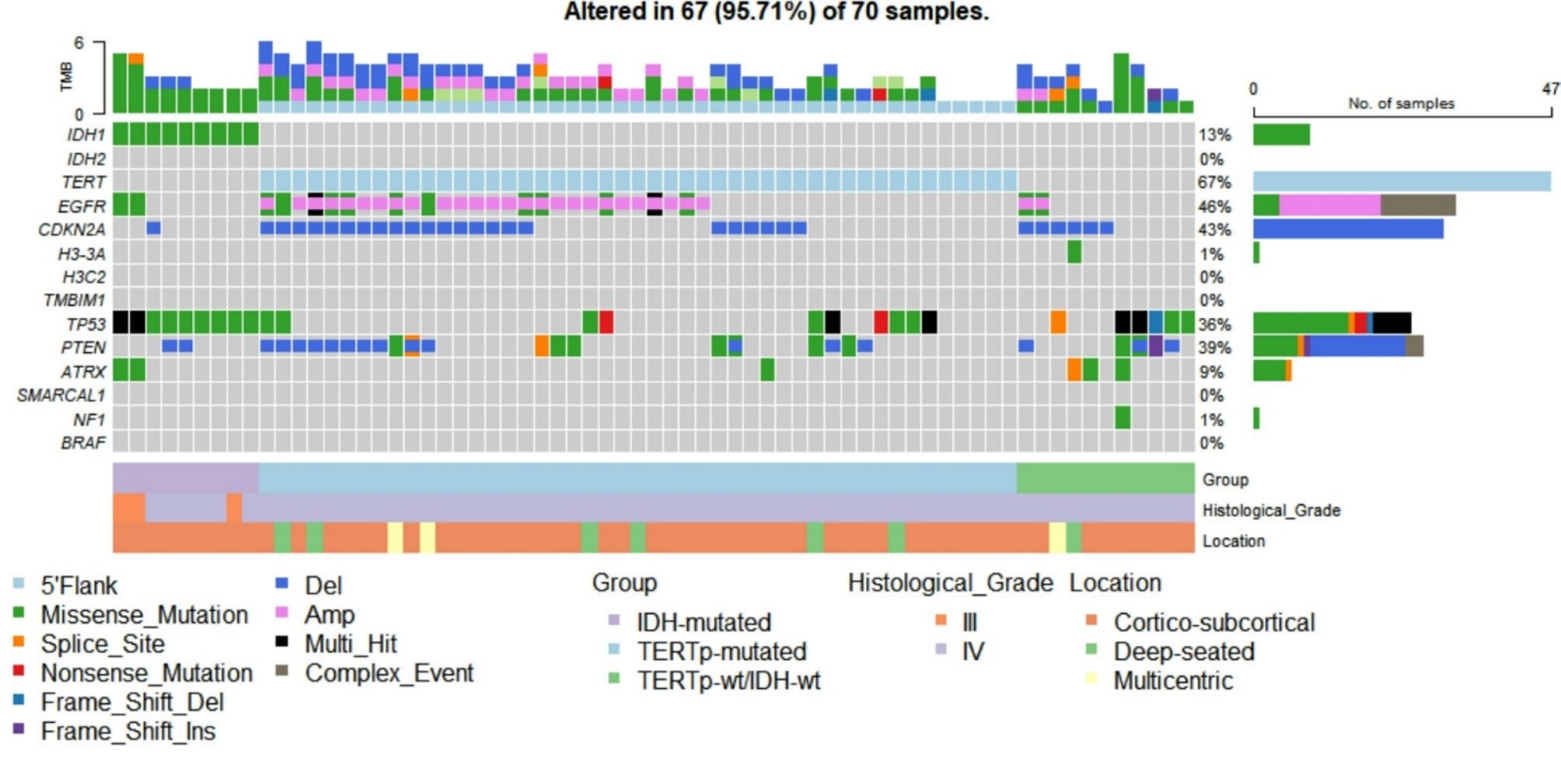
Oncoplot displaying genetic alterations in 14 genes included in our sequencing panel. Nex-generation sequencing results in 70 patients with high-grade astrocytoma are shown. Groups with different clinical outcomes, histological grade and tumor location for each patient are displayed below. All the multi-hit alterations in TERT promoter gene included the canonical mutations 113 and 135. *Multi-hit* 2 or more variants were identified, *Complex event* A copy number variation and another genomic alteration occurred simultaneously, *Amp* Gene amplification, *Del* Gene deletion, *IDH* Isocitrate Dehydrogenase, *TERTp* Telomerase Reverse Transcriptase Promoter, *TMB* Tumor mutational burden.

We sequenced key glioma-related genes currently used in molecular classification (*IDH1*, *IDH2*, *H3F3A*, *H3C2*, *EGFR*, *CDKN2A*, *ATRX*, *SMARCAL1*, *BRAF*, *NF1*, *TP53*, *TERTp* and *PTEN*). Overall, 60 unique variants were identified in 66 of the 70 patients (Fig. 1**and supplementary Table ****2**). Of the 70 patients, 67 showed genetic alterations when considering CNVs. The sequencing depths for each variant are shown in **supplementary Table 3** for all the samples. Our findings and comparisons with those of other populations for specific genes are detailed in the following sections.

### Mutations included in the WHO classification of CNS tumors

*IDH* is among the most relevant genetic markers currently in use for the classification of astrocytic tumors, and when mutated, it defines the recently termed *IDH-mutant astrocytoma*, which can be categorized as grade 2, 3, or 4 depending on additional histological and/or molecular criteria. This differentiation distinguishes IDH-mutated tumors from glioblastomas, which require *IDH*-wild-type confirmation. In our patient cohort, we found that ~ 13% of patients had *IDH1* mutations (9 out of 70): 8 had the most frequent R132H mutation, whereas 1 had the least common R132S mutation. The clinical characteristics of these patients were similar to those reported in other cohorts^20,21^significantly affecting younger subjects than those carrying *IDH*-wildtype alleles (49 years old (range, 30–76) vs. 59 years old (range, 24–75)). When the subgroup of tumors histologically graded as grade 4 was assessed, the frequency of *IDH1* mutations was 8.5% (6 out of 67), which was comparable to other cohorts^5^. As expected, due to their lower frequency^20^no *IDH2* mutations were identified in our cohort. Consistent with previous reports^21^none of the patients with *IDH1* mutations exhibited concomitant *TERTp* mutations.

Immunohistochemistry (IHC) for IDH1-R132H was routinely performed in patients diagnosed after 2017 (*n* = 38), showing a direct correlation with the sequencing results, with all six cases that had a positive R132 IHC showing a corresponding *IDH1* mutation. Interestingly, although the mean age of the nine patients carrying *IDH1* mutations was 49 years, two of these patients were older than 55 years, which is the most common cut-off for recommending sequencing in cases of negative R132H IHC^13^. The patient who carried the R132S mutation was older than 55 years and could not be accurately diagnosed using IHC, highlighting the relevance of using genomic techniques to evaluate *IDH1* mutations in astrocytoma patients.

Less common mutations that define specific entities according to the current diagnostic criteria are those in the *H3* gene family, including *H3F3A* and *HIST1H3B* genes^22^. In our cohort, we identified only one patient with an *H3* mutation who developed a thalamic tumor and carried the most frequent *H3F3A* mutation (K28M, rs1057519903), consistent with a specific entity, a *diffuse midline glioma*,* H3 K27-altered*. No *H3F3A* G35 or *HIST1H3B* were observed in our patients. Consistent with international cohorts^23,24^our findings indicate that adult patients with tumors located in different supratentorial regions have a low frequency of *H3* gene mutations.

In addition to the entity-defining markers, we evaluated the frequency of three genomic alterations that are currently considered in the grading of diffuse astrocytomas: *TERTp* mutations, *EGFR* amplification (molecular criteria for grade 4 glioblastoma), and *CDKN2A* deletion (molecular criterion for a grade 4 *IDH*-mutant astrocytoma). *TERTp* mutations typically occur in 70–80% of primary glioblastoma cases^25^. These mutations are closely associated with increased *TERT* expression, resulting in the preservation of telomeres, which play a pivotal role in promoting gliomagenesis^5^. We evaluated two canonical *TERTp* hotspots where mutations normally occur^25^. We found that 47 out of 70 patients (67.1%) carried one of these mutations, with 37 patients presented *TERTp* mutations at position g.chr5:1295113G > A and 10 patients with the less frequent g.chr5:1295135G > A mutation. These results are consistent with those of previous studies on European, North American, and Asian populations^26–28^. *EGFR* amplification is a recognized molecular hallmark of HGA. In our cohort, we found that 28 patients (40%) exhibited a significant increase in *EGFR* gene copy numbers. *EGFR* amplification was present only in *IDH*-wildtype tumors and was more frequent in *TERTp*-mutated cases (55.3% vs. 14.3%; *p* < 0.01). In contrast, *CDKN2A* deletion has been increasingly recognized as a prognostic marker in *IDH*-mutant astrocytomas^29^. *CDKN2A* partial or total deletions were present in 30 patients (42.9%). Only one patient with *CDKN2A* deletion also harbored an *IDH1* mutation. The frequency distribution and co-existence of *TERTp* and *IDH* mutations reported here are consistent with the results obtained in other brain cancer cohorts^30^.

Regarding grading changes based on molecular markers, all patients with *IDH*-wildtype tumors carrying *TERTp* mutations and/or *EGFR* amplification, as well as those with *IDH*-mutant tumors carrying *CDKN2A* deletions, were already classified as grade 4 based on histological features. Therefore, no grading adjustments were made based on these findings.

### Additional mutations related to HGA progression

*TP53* and *PTEN* genomic alterations are among the most prevalent mutations in gliomas, and multiple targeted therapies focusing on these genes have been evaluated over the last few years^31,32^. We sequenced the genomic regions containing all the previously described mutations in *TP53* and *PTEN* in glioblastomas and found a similar mutation rate for both genes compared to other cohorts^33,34^. Specifically, 25 out of 70 patients (allelic frequency (AF): 35.7%) harbored a *TP53* mutation, three of which had not been previously reported in astrocytoma patients (g.chr17:7669615delT (AF: 45% SIFT indel: neutral effect), g.chr17:7674926 C > T (rs587778719; AF: 10.6%; SIFT: tolerated; Polyphen-2: benign), and g.chr17:7674966delC (AF: 84.4%; SIFT indel: damaging). On the other hand, *PTEN* alterations were found in 29 out of 70 patients (41.4%), with partial or total deletions present in 18 patients, while single nucleotide variants were present in 13 patients. Out Of 11 unique *PTEN* variants identified, two corresponded to novel variants in astrocytoma patients (g.chr10:87925553 A > G (AF: 43.6%; SIFT: deleterious; Polyphen-2: probably damaging) and g.chr10:87933224_87933225insG (AF: 43.7% SIFT indel: damaging). Thus, novel *PTEN* (frameshift deletion g.chr17:7674966delC) and *TP53* variants (missense mutation g.chr10:87925553 A > G and frameshift insertion g.chr10:87933224_87933225insG) with predicted damaging effects were identified in our study, expanding the known mutations in these genes in patients with astrocytomas. However, each of these variants was only present in one patient, and four out of five carried an allelic frequency that suggests a somatic origin, therefore, it is unlikely that they constitute a specific hallmark of the Latin American population.

*ATRX* and *SMARCAL1* mutations are linked to telomerase-independent telomere maintenance in HGA^35,36^. While *ATRX* mutations are typically present in approximately 20% of HGA^36^our study identified a lower-than-expected frequency of 10% (7 out of 70) (*p* < 0.01), and none of the commonly reported genetic alterations were present in our cohort. However, numerous infrequent *ATRX* variants not covered by our sequencing design have been described. We screened the most common *SMARCAL1* cancer-associated mutations in our DNA sequencing panel, including Arg645Ser (R645S), Phe793del (del793), and Gly945fs*1 (945 fs) mutations, which have been previously described in up to 20% of *TERTp*-wt/*IDH*-wt tumors^37^. No *SMARCAL1* mutations were detected in our cohort.

Regarding *NF1*, previous cohorts of glioblastoma (TCGA pan-cancer atlas glioblastoma database, MSKCC dataset) have reported approximately a 13% mutation rate for this gene^38^which is significantly higher than we observed in our cohort (*p* < 0.01). Interestingly, although our panel design covered most of the commonly reported variants, none of these variants were present in our cohort, suggesting that *NF1* mutations are less frequent compared to other populations and/or are located in different genomic regions. Instead, we identified one variant of uncertain significance (rs2151559381) present in one patient, which has not been previously reported in patients with astrocytoma.

Finally, we examined the *BRAF* gene for the classic V600E mutation, which is traditionally associated with epithelioid features of glioblastoma^39^. Despite adequate coverage, sequencing revealed no *BRAF* mutations in our cohort.

### Survival analyses—association with relevant genes and clinical data

**Fig. 2:**
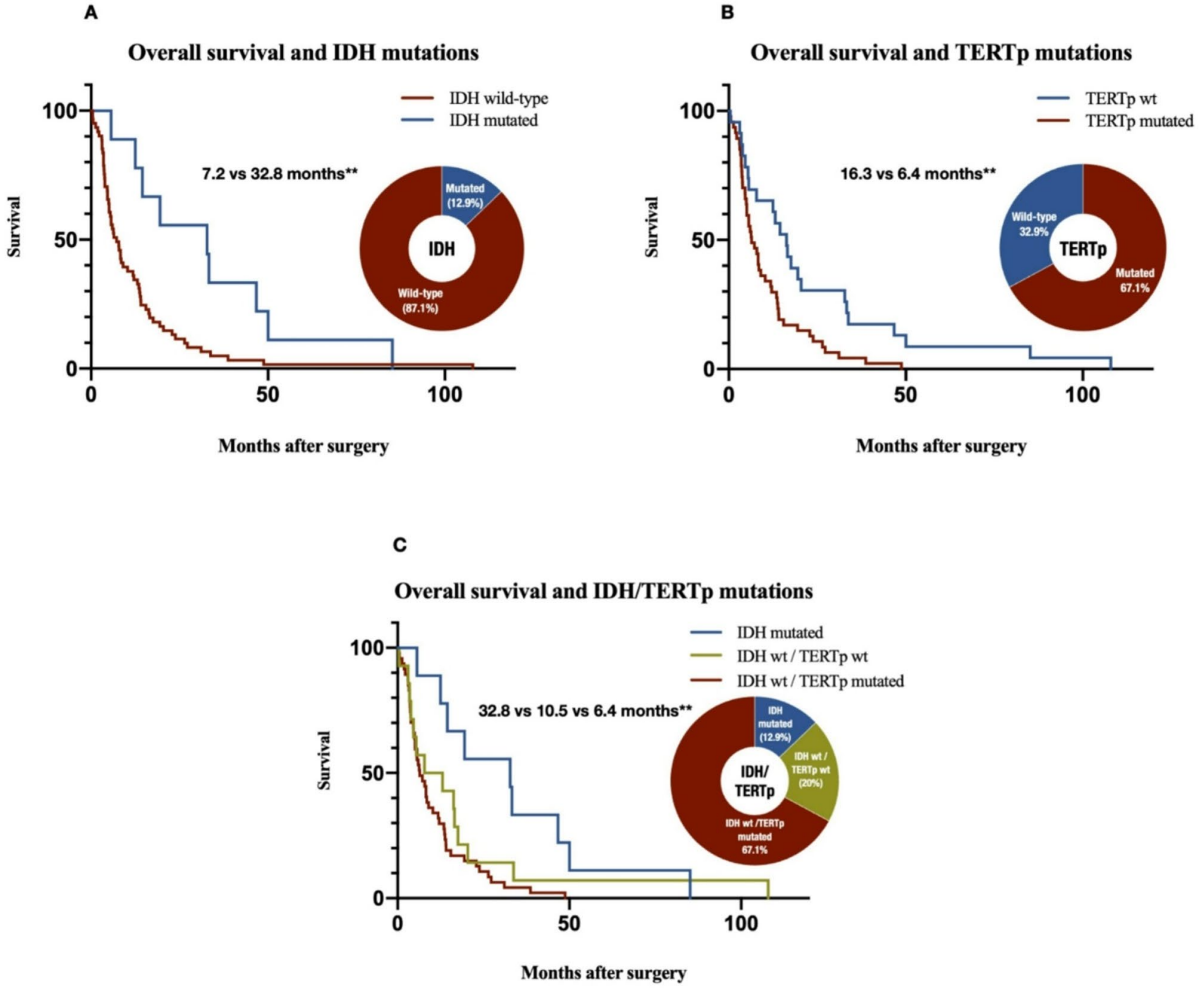
Stratified overall survival curves. Stratified Kaplan-Meier curves and distribution of mutation status are presented for (**A**) IDH mutations, (**B**) TERTp mutations and (**C**) a combined classification using IDH and TERTp mutations. Log-rank test was performed to compare curves, and double asterisks indicate *p*-values < 0.01. *IDH* Isocitrate Dehydrogenase, *TERTp* telomerase reverse transcriptase promoter.

Next, we compared the overall survival curves between patient groups defined by the presence of *IDH* mutations, *TERTp* mutations, or a combination of both, as the prognostic significance of these markers has been well documented^26,27^. In our study, *IDH* mutations were associated with significantly longer overall survival than *IDH* wildtype patients (32.8 vs. 7.2 months; *p* < 0.01; Fig. 2A), while patients with *TERTp* mutations had lower overall survival than those without *TERTp* mutations (6.4 vs. 16.3 months; *p* < 0.01; Fig. 2B). Our analysis revealed that *EGFR*, *TP53*, *PTEN*, and *CDKN2A* were not associated with a significant difference in overall survival (**Suppl. Figure 1**), which is consistent with previous reports^40^. Moreover, comparing survival curves based on *TERTp* mutations in IDH wildtype patients resulted in sustained survival differences (10.5 vs. 6.4 months; mantel-cox and logrank test for trend *p* < 0.01; Fig. 2C), highlighting the additive role of these mutations in predicting patient survival outcomes.

Regression analyses were conducted to evaluate the prognostic value of different clinical and molecular determinants in IDH wildtype tumors. We included all variables with univariate *p*-values < 0.25 in a multivariate analysis. The coefficients, confidence intervals, and significance values for univariate and multivariate analyses of each prognostic factor are shown in Table 2.

**Table 2:** Univariate and multivariate Cox regression models for clinical and genetic variables with a likely prognostic value for overall survival in *IDH* wildtype Astrocytoma patients (*n* = 61).

Predictive variable	Univariate						Multivariate					
	HR	Std. Err	z	p value	[95% CI]		HR	Std. Err	z	p value	[95% CI]	
Sex	0.77	0.20	− 0.97	0.33	0.46	1.30						
Age	1.02	0.01	1.23	0.22*	0.99	1.04	1.026	0.0136	1.93	0.054	0.9996	1.0531
Hypertension	1.34	0.36	1.10	0.27	0.80	2.26						
Diabetes	1.04	0.38	0.11	0.91	0.51	2.14						
Smoking	1.02	0.33	0.06	0.95	0.54	1.94						
Tumor location†	1.41	0.38	1.27	0.21*	0.83	2.39				0.77		
Favourable postoperative KPS (90–100)	0.54	0.16	− 2.10	0.04*	0.31	0.96				0.61		
Total vs. subtotal resection	0.35	0.10	− 3.65	< 0.01*	0.20	0.62	0.51	0.16	− 2.19	0.03**	0.27	0.93
Radiotherapy	0.21	0.07	− 4.57	< 0.01*	0.11	0.41	0.31	0.12	− 3.14	< 0.01**	0.15	0.64
Chemotherapy	0.15	0.07	− 4.08	< 0.01*	0.06	0.37	0.25	0.13	− 2.59	0.01**	0.09	0.72
*TERT* promoter mutation	1.41	0.45	1.08	0.28	0.76	2.62						
*EGFR* amplification	0.65	0.17	− 1.66	0.10*	0.39	1.08				0.13		
*CDKN2A*	0.82	0.21	− 0.75	0.46	0.50	1.37						

*KPS* Karnofsky Performance Score, *TERT* Telomerase Reverse Transcriptase, *CDKN2A* Cyclin-Dependent Kinase Inhibitor 2 A, *EGFR* Epidermal Growth Factor Receptor.

^†^Multicentric or deep-seated location versus cortico-subcortical location.

*p-values < 0.25 kept for multivariate analyses.

**p-values < 0.05 in multivariate analyses.

Significant values are in bold.

Our final multivariable regression model identified three factors capable of explaining overall survival in our HGA patients, all of them related to achieving optimal therapeutic goals: total versus subtotal resection, as well as radiotherapy and/or chemotherapy access. It is noteworthy that *TERTp* mutations exhibited significant interactions with other prognostic factors, such as age, postoperative Karnofsky Performance Status (KPS), and the utilization of radio- and chemotherapy (*p* < 0.01). These interactions could explain the lack of significance when analyzed together with these variables. For instance, patients who did not receive chemotherapy—often due to poorer clinical status or advanced age—were more likely to harbor TERTp mutations, limiting our ability to isolate the prognostic impact of this genetic alteration. Although this association is occasionally observed in clinical practice, it was likely exacerbated in our cohort due to limited access to chemotherapy. On the other hand, we did not include patients with *IDH*-mutant tumors in the regression analyses, to prevent any interaction effects that may influence the impact of other variables on survival.

## Discussion

Numerous studies have elucidated ethnic variations in the molecular markers associated with various cancer types in Latin American populations^41,42^. However, Hispanic and Latino populations have largely been underrepresented in HGA research^43^. While some reports have indicated disparities in both the incidence and prognosis of HGA among individuals of Latin American and non-Latino backgrounds^41,44^no comprehensive comparisons have been made regarding the molecular features of astrocytomas between Latin American and other populations.

Here, we conducted the first comprehensive molecular profiling of a Latin American HGA cohort to establish a basis for potential international comparisons. Our study on HGA in a Chilean population revealed findings comparable to those of other cohorts of North American, European, and Asian origin, suggesting a universal relevance of key molecular markers such as *IDH*, *TERTp*, *TP53*, and *PTEN* mutations as well as *EGFR* amplification and *CDKN2A* deletions^5,20,21,25,30^. Novel *TP53* and *PTEN* variants were identified and deemed likely pathogenic, based on in silico predictions. Mutations in the *ATRX* and *SMARCAL1* genes related to Alternative Lengthening of Telomeres (ALT) were observed less frequently than in other studies^36,37^. Nevertheless, the disparity in our results might be attributed, at least partially, to the design of our panel and partial coverage of some of the most commonly mutated regions within these genes. In addition, lower than expected mutation rates were found in *NF1*.

Given the recent updates in central nervous system tumor classification, the capacity to clinically identify mutations will become increasingly important in the upcoming years. For instance, in our series, *IDH* sequencing allowed for the re-categorization of a patient with negative IDH R132H IHC and an R172K mutation identified by NGS, moving from a glioblastoma to a grade 4 *IDH*-mutant astrocytoma. In addition, the relatively older age of patients with *IDH* mutations in this study suggests that the clinical use of DNA sequencing could be more relevant than that currently considered in patients over 55 years of age in our population. Similarly, our results re-categorized a patient with a *diffuse midline glioma*,* H3 K27-altered*, which is a pediatric-type tumor with worse prognosis than glioblastoma^22,24^.

Although the cost of sequencing has dramatically decreased over the last 15 years, the implementation and overall costs of the complete sequencing process remain very high for use in most developing countries, making a simplified targeted sequencing strategy, such as that used in this study, an attractive alternative. In this study, we optimized the conditions to reduce the risk of false negatives (DNA extraction from highly cellular tumor tissues, designing sequencing panels with high coverage depth, and setting low read number limits for known variants). However, standardized clinical implementation also requires the use of positive controls to establish the sensitivity and specificity of the sequencing strategy in a local context^45^.

### Study limitations

We acknowledge some limitations of the current study: (i) limited access to chemotherapy and radiotherapy has been a frequent occurrence in Latin American health systems during the last decade, and it generates a heterogeneous population to study the prognostic effect of genetic markers. (ii) the extent of resection was not systematically measured using immediate postoperative magnetic resonance imaging. (iii) O(6)-methylguanine-DNA methyltransferase (*MGMT*) promoter methylation was not determined, hence we could not evaluate its predictive value for temozolomide response^46^ in our cohort. (iv) Determination of gene copy number changes using amplicon sequencing as opposed to whole-genome sequencing has inherent limitations, mainly associated with coverage heterogeneity in different regions of interest^19^. These limitations were partially mitigated by bioinformatic processing of the data (see Methods). (v) Ancestry was determined by geography and self-report only, raising the possibility of an inadvertent mixture of genomic ancestries.

Our findings contribute to the global understanding of astrocytoma genetics and underscore the importance of inclusive research for advancing personalized medicine. Given that our study utilizes a targeted-sequencing approach, future research should expand upon these findings using more comprehensive methods and control data. This will help to further elucidate the genetic landscape of high-grade astrocytomas (HGA) in this population.

## Conclusion

Our HGA cohort from Latin America exhibited an incidence of *IDH*, *TERTp*, *PTEN*, and *TP53* mutations comparable to those reported in other studies with different ethnic origins. Conversely, *NF1*, *ATRX*, and *SMARCAL1* mutations had a lower frequency than expected. The correlation between *IDH* and *TERTp* mutations and patient survival indicated a similar association, as reported in previous studies in populations of non-Latino American, European, and Asian origin. This study contributes to the advancement of future international comparative studies, potential strategies for treating HGA and population-specific health strategies. Our findings highlight the multifaceted nature of HGA, underscoring its complex molecular origins and the distinctive genetic diversity that exists among various populations. We emphasize the importance of ensuring equitable access to advanced molecular diagnostics, shedding light on the need for and approaches to the widespread adoption of state-of-the-art technologies in healthcare systems.

## Electronic supplementary material

Below is the link to the electronic supplementary material.

Supplementary Material 1

Supplementary Material 2

## Data Availability

All raw sequencing data can be found on the Gene Expression Omnibus (GEO) website: https://www.ncbi.nlm.nih.gov/geo/query/acc.cgi?acc=GSE252870.
