# Supplementary material for: Characterization of the mutational status of glioblastoma and high-grade astrocytomas in a Latin American cohort
Source: Sci Rep. 2025 Oct 3;15:34485. doi: 10.1038/s41598-025-06129-z (PMC12494855; doi:10.1038/s41598-025-06129-z)
Supplement: Supplementary file 2 — Supplementary Material 2 [file 41598_2025_6129_MOESM2_ESM.docx]

**Supplementary Methods**

**Panel Design, Parallel Mass Sequencing and Bioinformatic Analysis**

Formalin-fixed paraffin-embedded tissue samples were obtained from the Pathology Service at the Asenjo Neurosurgery Institute. After neuropathologist confirmation of a tumor percentage greater than 70% in each area to be extracted, 3-6 sections of 10 microns were prepared from each paraffin block using the GeneJET FFPE DNA Purification Kit. No matched blood samples were available. The extracted DNA was quantified using fluorescence-based methods (Quant-iT™ PicoGreen™ dsDNA and/or Qubit™ dsDNA HS), resulting in an average concentration of 40 ng/µL.

Databases such as cbioportal, NCBI/Clinvar, and the Catalogue of Somatic Mutations in Cancer (COSMIC) were employed to determine the relevance and genomic coordinates of sites of interest. Amplicons were designed to encompass these mutations using the AmpliSeq™ for Illumina tool (Supplementary Table 1). This sequencing strategy included 339 amplicons with an average length of 134 base pairs (ranging from 125 to 140 base pairs) and covered 15,596 base pairs, including the following genes: *IDH1*, *IDH2*, *H3F3A*, *H3C2*, *EGFR*, *CDKN2A*, *ATRX*, *SMARCAL1*, *BRAF*, *TP53*, and *PTEN*. Because the promoter region of the *TERT* gene contains a high percentage of GC bases ^1^, a complementary sequencing strategy was designed to cover the region using 11 amplicons with an average length of 226 base pairs (ranging from 193 to 264 base pairs) that covered 2,492 base pairs. 1 amplicon was designed to cover the *TERT* promoter region, and the rest of the amplicons were designed to either give additional coverage for the most relevant hotspots or to assess additional regions of interest (Supplementary Table 1).

Library preparation and parallel mass sequencing were conducted at the Advanced Genomics Core of the Universidad Mayor. An AmpliSeq™ Custom DNA Panel kit and a TruSeq™ nano DNA kit from Illumina were used to prepare libraries for each sequencing strategy, respectively. For both AmpliSeq™ and TruSeq™ libraries, library generation efficiency was evaluated with different initial amounts of DNA to adjust the various stages of the process based on the specific sample and panel characteristics. Automated electrophoresis of the first libraries were performed for quality control and size estimation using the DNA 1000 kit on the Agilent 2100 Bioanalyzer system. All libraries were quantified by qPCR using the Illumina KAPA library quantification kit and were pooled at a concentration of 10 nM before sequencing. A Mid Output Kit v2.5 (150 cycles) was used on a NextSeq sequencer with 25% phi-X for all Ampliseq™ libraries, and a Micro Kit v2 (300 cycles) with 25% phi-X on a MiSeq sequencer was used for all TruSeq™ libraries.

*.fastQ files were generated from the raw sequencing data and analyzed using the fastQC software for quality control (https://www.bioinformatics.babraham.ac.uk/projects/fastqc/). Reads of low quality and adapter contamination were removed using the trim_galore tool ^2^. Processed reads were aligned to the GRCh38 reference genome using the Bowtie2 tool ^3^. The aligned *.sam results were then converted to *.bam files using the samtools application for subsequent analysis. Somatic mutations were called according to GATK recommendations ^4–6^, including additional considerations for amplicon panels, as described below. Bam files were analyzed with MarkDuplicatesSpark and BaseRecalibrator, using gnomadV2 database ^7^, to finally recalibrate with ApplyBQSR to meet GATK standards. Somatic variants were detected using Mutect2 ^8^, only on target regions. To identify and discard artifactual variants, GetPileupSummaries with CalculateContamination, LearnReadsOrientation and a panel of normal were used, Additionally, the Integrative Genomics Viewer (IGV 2.10) was used to identify and correct potential errors. Finally, we used FilterMutectCalls to recover clustered events and to discard variants with an allelic frequency <0.05 or <10 reads supporting the variant.

Variants in vcf files were annotated with Funcotator and reports were generated in vcf and maf files ^4^. All genomic coordinates are mapped to the GRCh 38 assembly. Posterior analysis was performed with Variant Effect Predictor ^9^ to predict pathogenicity. Novel variants and variants not previously reported in gliomas that are predicted to result in single amino acid substitutions were subsequently evaluated using Sorting Intolerant From tolerant (SIFT) and polymorphism phenotyping-2 (PolyPhen-2) to predict the effect of the change on protein function ^10,11^ while variants that resulted in frameshifts were evaluated using SIFT indel ^12^. Maftools R package was used to prepare oncoplots ^13^.

Copy number analysis of *EGFR*, *CDKN2A* and *PTEN* was performed using the R package CNVPanelizer ^14^, which normalizes the number of reads for the gene of interest based on the total reads for each sample, followed by a resampling algorithm (bootstrapping with replacement, n=10,000) to mitigate the effect of coverage heterogeneity in data interpretation. The fold change of *EGFR*, *CDKN2A* and *PTEN* in relation to 5 control samples were determined, using coverage information from 136 *EGFR* amplicons, 21 *CDKN2A* amplicons, 46 *PTEN* amplicons and 120 amplicons outside the regions of interest (*ATRX*, *NF1*, *BRAF*, and *TP53*). The statistical significance of the results was assessed using the Bonferroni test, and only CNVs that were significant are reported.

**Statistical analysis**

Associations between categorical variables were assessed by the χ2 test. Kaplan–Meier estimates and log-rank testing and log-rank testing for multiple groups were performed for survival curves analyses. Proportional hazards Cox regressions were used to examine the relationship between predictor variables and overall survival, and a stepwise forward selection was used when multiple predictors were analyzed. To determine whether the observed mutation frequencies in our cohort were significantly lower than the expected rates reported in the literature, we performed one-sample proportion Z-tests and a *p* value <0.05 was considered statistically significant.

**Supplementary results**

**Sequencing metrics**

A Q-score greater than Q30 was calculated for over 90% of the sequencing reads and were subsequently used for variant calling. The percentage of reads on-target exceeded 90% in all patients.

**Supplementary References**

1. Chiba K, Johnson JZ, Vogan JM, Wagner T, Boyle JM, Hockemeyer D. Cancer-associated tert promoter mutations abrogate telomerase silencing. *Elife*. 2015;4(JULY 2015):1-20. doi:10.7554/ELIFE.07918

2. Bolger AM, Lohse M, Usadel B. Trimmomatic: a flexible trimmer for Illumina sequence data. *Bioinformatics*. 2014;30(15):2114. doi:10.1093/BIOINFORMATICS/BTU170

3. Langmead B, Salzberg SL. Fast gapped-read alignment with Bowtie 2. *Nat Methods*. 2012;9(4):357-359. doi:10.1038/nmeth.1923

4. Depristo MA, Banks E, Poplin R, et al. A framework for variation discovery and genotyping using next-generation DNA sequencing data. *Nat Genet*. 2011;43(5):491-501. doi:10.1038/NG.806

5. McKenna A, Hanna M, Banks E, et al. The Genome Analysis Toolkit: a MapReduce framework for analyzing next-generation DNA sequencing data. *Genome Res*. 2010;20(9):1297-1303. doi:10.1101/GR.107524.110

6. Van der Auwera GA, Carneiro MO, Hartl C, et al. From FastQ data to high confidence variant calls: the Genome Analysis Toolkit best practices pipeline. *Curr Protoc Bioinformatics*. 2013;43(1110). doi:10.1002/0471250953.BI1110S43

7. Gudmundsson S, Singer-Berk M, Watts NA, et al. Variant interpretation using population databases: Lessons from gnomAD. *Hum Mutat*. 2022;43(8):1012-1030. doi:10.1002/HUMU.24309

8. Benjamin D, Sato T, Cibulskis K, Getz G, Stewart C, Lichtenstein L. Calling Somatic SNVs and Indels with Mutect2. *bioRxiv*. Published online December 2, 2019:861054. doi:10.1101/861054

9. McLaren W, Gil L, Hunt SE, et al. The Ensembl Variant Effect Predictor. *Genome Biol*. 2016;17(1). doi:10.1186/S13059-016-0974-4

10. Kumar P, Henikoff S, Ng PC. Predicting the effects of coding non-synonymous variants on protein function using the SIFT algorithm. *Nature Protocols 2009 4:7*. 2009;4(7):1073-1081. doi:10.1038/nprot.2009.86

11. Adzhubei IA, Schmidt S, Peshkin L, et al. A method and server for predicting damaging missense mutations. *Nature Methods 2010 7:4*. 2010;7(4):248-249. doi:10.1038/nmeth0410-248

12. Hu J, Ng PC. Predicting the effects of frameshifting indels. *Genome Biol*. 2012;13(2):R9. doi:10.1186/GB-2012-13-2-R9

13. Mayakonda A, Lin DC, Assenov Y, Plass C, Koeffler HP. Maftools: efficient and comprehensive analysis of somatic variants in cancer. *Genome Res*. 2018;28(11):1747-1756. doi:10.1101/GR.239244.118

14. Oliveira C, Wolf T. CNVPanelizer: Reliable CNV detection in targeted sequencing applications. R package version 1.23.0. Published online 2020:1-8. Accessed September 5, 2021. https://www.bioconductor.org/packages/devel/bioc/vignettes/CNVPanelizer/inst/doc/CNVPanelizer.pdf

**Supplementary Legends**

**Supplementary Figure 1. Stratified overall survival curves.** Stratified Kaplan-Meier curves and distribution of mutation status are presented for (A) *EGFR* amplification, (B) *CDKN2A* deletion (C) *TP53* mutations and (D) *PTEN* mutations. Log-rank test was performed to compare curves, and no significant differences were found between groups.

**Supplementary Table 1. Target regions of the panel design.** The target coordinates for the Ampliseq™ and Truseq™ sequencing strategies are listed. The Ampliseq™ design included 339 amplicons with an average length of 134 bp (ranging from 125 to 140 bp) and covered 15,596 bp, including the following genes: *IDH1*, *IDH2*, *H3F3A*, *H3C2*, *EGFR*, *CDKN2A*, *ATRX*, *SMARCAL1*, *BRAF*, *TP53*, and *PTEN*. The Truseq™ design included 11 amplicons with an average length of 226 bp (ranging from 193 to 264 bp) that covered 2,492 bp, including the following genes: *TERT* promoter, *H3F3A*, *IDH1*, *IDH2*, *SMARCAL1*, and *CDKN2A*.

**Supplementary Table 2. Summary of genomic variants.** Variant coordinates, classification, allelic frequencies, protein changes, and samples presenting each variant are provided. 60 unique variants were identified in 66 of the 70 patients.

**Supplementary Table 3. Summary of genomic variants coverage.** The sequencing depths (number of sequencing reads) for each variant are shown for all the samples. The average and median coverage for each variant are also provided.
